# Changes in the levels of free sialic acid during ex vivo lung perfusion do not correlate with pulmonary function. Experimental model

**DOI:** 10.1186/s12890-023-02619-w

**Published:** 2023-09-04

**Authors:** Claudia Hernández-Jiménez, Javier Martínez-Cortés, J. Raúl Olmos-Zuñiga, Rogelio Jasso-Victoria, María Teresa López-Pérez, Néstor Emmanuel Díaz-Martínez, Marcelino Alonso-Gómez, Axel Edmundo Guzmán-Cedillo, Matilde Baltazares-Lipp, Miguel Gaxiola-Gaxiola, Adriana Méndez-Bernal, Adrián Polo-Jeréz, Juan Carlos Vázquez-Minero, Oscar Hernández-Pérez, Christopher O. Fernández-Solís

**Affiliations:** 1 Department of Surgery Research of National Institute of Respiratory Diseases Ismael Cosío Villegas, Mexico City, Mexico; 2 Experimental Lung Transplant Unit of National Institute of Respiratory Diseases Ismael Cosío Villegas, Mexico City, Mexico; 3 Nursing Research Coordination of National Institute of Respiratory Diseases Ismael Cosío Villegas, Mexico City, Mexico; 4 Department of Medical and Pharmaceutical Biotechnology, Center for Research and Assistance in Technology and Design of the State of Jalisco, Jalisco, Mexico; 5 Laboratory of Morphology of National Institute of Respiratory Diseases Ismael Cosío Villegas, Mexico City, Mexico; 6 Electron Microscopy Unit, Faculty of Veterinary Medicine and Zootechnics, National Autonomous University of Mexico, Mexico City, Mexico; 7 Cardiothoracic Surgery Service of National Institute of Respiratory Diseases Ismael Cosío Villegas, Mexico City, Mexico; 8 Department of Physiology, School of Medicine, National Autonomous University of Mexico, Mexico City, Mexico

**Keywords:** Organ preservation solution, Experimental model, Ex vivo lung perfusion, N-Acetylneuraminic acid

## Abstract

**Background:**

Ex vivo lung perfusion (EVLP) constitutes a tool with great research potential due to its advantages over in vivo and in vitro models. Despite its important contribution to lung reconditioning, this technique has the disadvantage of incurring high costs and can induce pulmonary endothelial injury through perfusion and ventilation. The pulmonary endothelium is made up of endothelial glycocalyx (EG), a coating of proteoglycans (PG) on the luminal surface. PGs are glycoproteins linked to terminal sialic acids (Sia) that can affect homeostasis with responses leading to edema formation. This study evaluated the effect of two ex vivo perfusion solutions on lung function and endothelial injury.

**Methods:**

We divided ten landrace swine into two groups and subjected them to EVLP for 120 min: Group I (n = 5) was perfused with Steen® solution, and Group II (n = 5) was perfused with low-potassium dextran-albumin solution. Ventilatory mechanics, histology, gravimetry, and sialic acid concentrations were evaluated.

**Results:**

Both groups showed changes in pulmonary vascular resistance and ventilatory mechanics (p < 0.05, Student’s t-test). In addition, the lung injury severity score was better in Group I than in Group II (p < 0.05, Mann–Whitney U); and both groups exhibited a significant increase in Sia concentrations in the perfusate (p < 0.05 t-Student) and Sia immunohistochemical expression.

**Conclusions:**

Sia, as a product of EG disruption during EVLP, was found in all samples obtained in the system; however, the changes in its concentration showed no apparent correlation with lung function.

## Background

Ex vivo lung perfusion (EVLP) originated as a tool for offsetting the scarcity of organs for transplant. EVLP reconditions the lung by simulating a physiological environment through pulmonary perfusion and ventilation. Currently, the development of research protocols on EVLP is also aiming to use this platform to resolve crucial issues related not only to clinical areas but also to basic science [1, 2]. However, although it is a technique with great advantages in pulmonary reconditioning, it has the disadvantage of having a high cost and can induce injury to the pulmonary endothelium through perfusion and ventilation [3]. The luminal surface of the endothelium is covered by endothelial glycocalyx (EG) composed of proteoglycans (PG), glycoproteins bound to terminal sialic acids (Sia), glycosaminoglycans (GAG) and associated plasma proteins [4].

Currently, over 50 members have been identified in the Sia family with specific structural characteristics; among them, N-acetylneuraminic acid (Neu5Ac) andmariscal its hydroxylated form, N-Gluconeuraminic acid (Neu5Gc), have been found in mammals in large proportions [5]. An important regulator of endothelial function, Neu5Gc, plays a key role in the regulation of microvascular tone and permeability and maintaining the oncotic gradient through the endothelial barrier. It also assists in the adherence/migration of leucocytes and the inhibition of intravascular thrombosis, lipid metabolism, and inflammation, so the loss of its integrity due to endothelial damage affects homeostasis, leading to inflammatory responses and edema formation [6, 7]. Despite its fundamental role in the endothelium, EG has proven difficult to study because of its complex chemistry. However, recent investigations have shown the relevance of Sia and other components of GE in various pathologies; therefore, their presence may be an indicator of endothelial damage by lung perfusion during EVLP. Taking the above into account, the objective of this study was to determine the difference in lung injury parameters after EVLP with two different perfusion solutions, using sialic acid levels as an indicator of the integrity of the endothelial glycocalyx and analyzing its relationship with lung function during EVLP.

## Materials and methods

We performed our experiments in the Department of Surgery Research at the National Institute of Respiratory Diseases Ismael Cosío Villegas (*INER*). We conducted a prospective, longitudinal, randomized study of 10 healthy landrace pigs, regardless of sex, with weights ranging between 15 and 20 kg. Prior to the experiment, we confined the animals in individual cages with identical environmental conditions and provided them with water and food *ad libitum*. The Institutional Review Board approved the protocol (B09-17). We treated all animals in strict accordance with the ARRIVE Guidelines [8, 9].

### Study groups

All animals underwent cardiopulmonary block procurement and were divided as follows: Group I (n = 5): EVLP with gold standard Steen® solution; and Group II (n = 5): EVLP with low-potassium dextran-albumin solution (LPDA). The lungs of all swine were perfused ex vivo for a continuous 120-minute period, during which the parameters of lung function were assessed as described below.

### Donor procedures

All procedures were performed under general anesthesia. Induction was performed with tiletamine zolazepam (4 mg/kg, IM. Zoletil, Virbac, Carros, France) and propofol (4 mg/kg, IV. Recofol, PISA, Guadalajara, JAL, Mexico) and then maintained with isoflurane (Forane, Abbott Mexico S.A. de C.V., Mexico City, Mexico) and fentanyl (0.1 mg/kg, IV. Fentanest, Janssen- Cilag, Puebla, Mexico) as analgesic. The animals were ventilated with pulmonary protection strategies, and the hemodynamic, gasometric, and ventilation mechanics parameters were assessed; the cardiac output was determined using the thermodilution method (Hemodynamic Profile CARESCAPE B650 (General Electric Company ©, Finland)). Recruitment maneuvers are performed to a pulmonary artery wedge pressure (PawP) of 25 cm H_2_O. Subsequently, cardiopulmonary block procurement was performed with the technique described by Mariscal, et al. [10]. All animals were euthanized with an overdose of sodium pentobarbital (150 mg/kg/IV, Anestesal, Pfizer, S.A. de C.V., Guadalajara, Mexico) upon completion of the donor operation.

### Preparation of lungs for EVLP

A funnel-shaped cannula (Vitrolife, Göteborg, Sweden) was sewn to the left atrial cuff, a cannula (Vitrolife, Göteborg, Sweden) was secured into the pulmonary artery (PA), and a 7 − 0 endotracheal tube with the balloon removed was secured into the trachea. The EVLP circuit consisted of extracorporeal circulation with a neonatal reservoir VHK 1100 (Maquet Getinge Group, Germany) and a neonatal oxygenator (Quadrox-i Maquet, Germany) connected to a pump CDL-10,140 (Gambro, USA). [11, 12]

The lungs were transferred to an XVIVO chamber (XVIVO, Göteborg, Sweden), and retrograde flow was initiated through the left atrium to deair the pulmonary vasculature and flush any remaining clot. The PA cannula was then connected, and antegrade flow began at 0.1 L/min. EVLP was performed using acellular Steen® solution (XVIVO, Göteborg, Sweden), a commercially available preservative solution designed for ex vivo lung assessment, supplemented with 10,000 IU heparin (APP Pharmaceuticals, Schaumburg, Ill, USA). The perfusate was slowly warmed to 37 °C during a 30-minute period as the flow was titrated up to the target of 40% of the estimated cardiac output.

When the perfusate reached 32 °C, ventilation was initiated with room air at a tidal volume of 6–8 mL/kg, a respiratory rate of 8 breaths/min, and a positive end-expiratory pressure (PEEP) of 5.0 cm H2O. Recruitment maneuvers to a maximum of 25 cm H_2_O are used to recruit regions of lung atelectasis [12]. After initiation of ventilation, a mixture of 6% oxygen, 8% carbon dioxide, and 86% nitrogen was infused into the membrane oxygenator to deoxygenate the PA perfusate and allow for accurate measurement of lung oxygenation capability. Every hour after EVLP initiation, PaO_2_ was evaluated with a fraction of inspired oxygen (FiO_2_) at 21%, and then, the lungs were ventilated with (FiO_2_) at 100% for 10 min, and another sample of the perfusate was taken from the left atrial return for gas analysis [12–14].

### Assessment

#### Lung function

The study was conducted for 2 h. The hemodynamic, gasometric, and ventilation mechanics parameters were assessed (Hemodynamic Profile CARESCAPE B650 (General Electric Company ©, Finland)). Gas exchange was evaluated by measuring the relationship between arterial pressure of oxygen and the fraction of inspired oxygen (PaFiO_2_). Arterial oxygen partial pressure (PaO2), arterial carbon dioxide partial pressure (PaCO2), bicarbonate (HCO3), glucose and lactate (ABL 800 Flex Analyzer [Radiometer, Brønshøj, Denmark) were also determined. We used the perfusate returning from the lung for comparison since it would represent the lung microenvironment. Samples of perfusate were taken after 10 min of exposure to fractions of inspired oxygen (FiO_2_) at 1.0. Static (Cstat) and dynamic (Cdyn) lung compliance, airway resistance (Raw), peak airway pressure (Ppic), and mean airway pressure (Paw) were measured at the start and end times (Avea™ VIASYSTM Health care, USA); start time refers to the moment each lung was successfully rewarmed to 37 °C [11, 12] at the beginning of EVLP, while end time denotes 120 min of EVLP.

### Microscopic assessment

Biopsies were taken at the beginning and end of EVLP; samples were obtained from right lobes in all experiments with areas of the lung whose macroscopic appearance presented lesions, in an attempt to cover the transition areas between sites with areas of normal appearance.

The samples were fixed in 10% phosphate-buffered formalin for 24 h and paraffin embedded, then they were sectioned *and* stained with hematoxylin and eosin (HE). The most prominent features observed in the lungs were blindly assessed by a pathologist with a scoring system, as they searched for evidence of cell infiltration in the lungs (neutrophils, macrophages, and lymphocytes) and edema. The severity of the findings was graded on a scale from 1 (absent) to 3 (severe) [15].

### Transmission electron microscopy

Transmission electron tissue samples were fixed in 2.5% glutaraldehyde, buffered in 0.1 M Na-P-buffer overnight, washed 3 times in 0.1 M buffer, postfixed in 1% osmium tetroxide and dehydrated in ascending concentrations of acetone followed by infiltration in epoxy resin (Epon 812, Electron Microscopy Sciences, Hatfield, PA USA).

At least 150 nm toluidine blue (Electron Microscopy Sciences, PA USA)-stained semithin sections per localization were produced. Representative areas were trimmed, and 90 nm lead citrate and uranyl acetate (Electron Microscopy Sciences, PA USA) contrasted ultrathin sections were produced and subsequently viewed under an electron microscope at 60 kV (Jeol 1010, Massachusetts, USA).

### Determination of pulmonary edema

The lung tissues were weighed and dried in an oven between 60 and 65 °C to constant weight. Finally, the lung weight gain was calculated with the following formula: ΔPP = (PH – PS)/PS, where ΔPP is the lung weight gain, PH is the final lung weight, and PS is the initial lung weight.

### Immunohistochemical expression

Tissue was deparaffinized for 20 min at 60 °C; Diva Decloaker, 20X (Biocare medical, CA, USA) and Decloaking chamber™ NXGEN were used for recovery. The sections were incubated with primary antibody (Polyclonal Antibody to Sialic Acid, abx 100,414, abbexa Ltd. Cambridge, UK), and PECAM-1 (Polyclonal Antibody Abbiotec 250,590) diluted in blocking serum and incubated for 48 h at 4 °C, then washed 3 times with TBST, for 3 min each. They were then incubated with MACH 2 Double Stain 1 polymer (Biocare Medical MRCT523, CA, USA) for 30 min and washed with TBST. Diaminobenzidine DAB (Biocare Medical, CA, USA) was used for development. A negative control, which had no primary antibody, was performed in all groups, and salivary gland tissue was used as control tissue. IHC quantification of sialic acid was performed with ImageJ software (https://www.rsbweb.nih.gov/ij) developed by the National Institute of Health (NIH), using the IHC Profiler plug-in for the quantitative analysis of immunohistochemistry samples [16].

For evaluation of CD31/PECAM-1 immunohistochemical staining, slides were observed under a light microscope, and positivity was determined in ten microvessels. [17]

### Immunofluorescence

Lectin analysis was performed using the fluorescein-labeled lectins Sambucus nigra agglutinin (SNA), which primarily detects 6-linked sialic acids, and Maackia amurensis agglutinin (MAA), which primarily identifies 3-linked sialic acids. Both fluorescein isothiocyanate (FITC) and tetramethyl rhodamine isothiocyanate (TRITC) were used as fluorochromes and purchased from EY Laboratories (San Mateo, California). The tissues were sectioned at 5 μm and deparaffinized. Control sections did not undergo the retrieval procedure. Single fluorescent studies were performed as follows. The sections were microwaved in 95 °C citrate buffer pH 6.0 for 15 min, washed with 0.05 M Tris-buffered saline (TBS), pH 7.6, and then incubated with either 1/100 FITC-conjugated SNA (EY Laboratories) or 1/100 FITC-conjugated MAA (EY Laboratories) for 1 h at room temperature in the dark. The sections were washed with TBS 3 times for 5 min each, and the nuclei were stained with 5 µg/ml DAPI for 4 min, followed by three washes with TBS for 5 min each and mounted with Fluoro Care Anti-Fade Mountant (Biocare Medical, CA, EUA). Fluorescence examination was performed with a ZEISS Axio Vert. A1 microscope.

### Quantification of Sia

We utilized the Sia (NANA) Colorimetric Assay Kit BioVision K566-100 (Mountain View, CA, USA) to quantify the levels of free Sia at the beginning and end of the EVLP process in bronchoalveolar lavage (BAL) through a flexible bronchoscope (Olympus®, Tokio, Japan). To assess its concentration in the tissue, one gram of material was taken and homogenized (Polytron® pt 2500 e kinematica, Lucerna, Suiza) with phosphate buffered saline (PBS) on ice. We then centrifuged the material in tubes (Eppendorf Safe Lock®, CA, USA) at 13,500 rpm, transferred the supernatant into sterile polypropylene cyrovials (nalgene®, Massachusetts, USA), and stored it at -80 °C until evaluation. Finally, we used the perfusate returned from the lung.

### Statistical analysis

Paired samples, start-end of perfusion, were compared with Student’s t-test to identify differences between samples in each group, and unpaired samples were used to identify differences between groups. The nonparametric Mann‒Whitney test or Wilcoxon signed rank test was used to compare significant differences between the two groups, and Friedman’s two-way analysis of variance was conducted using the rank of related samples. Pearson’s correlation (r) was used to measure the linear dependence between lung function and Sia levels. SPSS 19.0 statistical software (SPSS Inc., Chicago, USA) was used, and p values of p < 0.05 were considered as significant.

## Results

We performed EVLP on all lungs for 120 min, and all parameters were within normal values for swine. We observed changes only in the following parameters:

Functional outcomes. PVR increased in both groups compared to their initial values (p > 0.05), and when comparing between groups, we found differences (p = 0.002). Table 1.

In comparing arterial oxygen partial pressure (PaO_2_)/inspired oxygen fraction (FiO_2_) ratios during EVLP, we observed an improvement in both groups (p > 0.05) but detected no significant differences between them (p > 0.05). Table 1.

The partial pressure of carbon dioxide (PCO_2_) diminished in both groups compared to their initial values (p < 0.05) for the Steen® group and (p > 0.05) for the LPDA group, with differences emerging between groups at specific study times (p = 0.001) Table 1. HCO_3_ showed differences between the Steen® and LPDA groups at the start (p = 0.0001) and end (p = 0.001) of EVLP. Table 1.

During EVLP, static (Cstat) and dynamic (Cdyn) lung compliance decreased in the LPDA group compared to the initial values (p > 0.05), with differences (p = 0.006) emerging between groups (Table 1). Raw, Ppic and Paw demonstrated a slight increase in both groups when compared to initial values (p > 0.05), and when compared between groups, only Paw was different (p = 0.001). Table 1.

**Table 1:** Pulmonary physiological parameters

	Start			End			
	*Steen*	*LPDA*	*P value* ^*1*^	*Steen*	*LPDA*	*P value* ^*2*^	*P value* ^*3*^
**FiO**_**2**_ 1.0							
**PVR dynas·s·cm** ^**− 5**^	491.04 ± 123.79	1040.82 ± 77.77	P = 0.178	602.54 ± 251.07	1040.80 ± 101.81	P = 1.000	P = 0.002
**PO**_**2**_ **perfusate mmHg**	445.80 ± 49.6	443.20 ± 90.9	P = 0.382	470.80 ± 50	487 ± 112.7	P = 0.802	P = 0.093
**PC0**_**2**_ **perfusate mmHg**	20.40 ± 4.66	32.18 ± 4.20	P = 0.445	18.38 ± 4.39	30.54 ± 2.14	P = 0.009	P = 0.001
**HCO**_**3**_ **perfusate mmol**	2.86 ± 0.65	7.98 ± 1.38	P = 0.35	2.64 ± 0.71	6.72 ± 1.63	P = 0.016	P = 0.001
**Glucose perfusate** **mmol/L**	2.64 ± 0.51	7.74 ± 1.86	P = 0.081	1.66 ± 0.48	5.66 ± 1.91	P = 0.043	P = 0.002
**Lactate perfusate mmol/L**	7.40 ± 1.12^+^	4.84 ± 2.35	P = 0.037	9.56 ± 2.06	7.22 ± 2.02	P = 0.047	P = 0.108
**Cstat ml/cmH**_**2**_ **O**	5.6 ± 1.14	11 ± 4.74	P = 0.374	5.4 ± 1.34	10 ± 2.44	P = 0.546	P = 0.006
**Cdyn ml/cmH**_**2**_ **O**	4.8 ± 0.83	12.2 ± 3.27	P = 0.52	4.8 ± 0.83	10.2 ± 4.6	P = 0.327	P = 0.040
**Raw cmH**_**2**_ **O/L/S**	26.82 ± 6.89	33.68 ± 4.05	P = 0.157	31.46 ± 6.50	34.74 ± 2.87	P = 0.366	P = 0.333
**Ppic** **cmH**_**2**_ **O**	19 ± 3.24	16.6 ± 1.51	P = 0.621	19.2 ± 3.19	19.4 ± 5.27	P = 0.231	P = 0.944
**Paw** **cmH**_**2**_ **O**	4.8 ± 0.83	7 ± 0	P = 0.374	5 ± 1	7.8 ± 0.83	P = 0.099	P = 0.001

Average ± SD. FiO_2_: inspired oxygen fraction, PVR: pulmonary vascular resistance, PaO_2_: arterial oxygen partial pressure, PaCO_2_: arterial carbon dioxide partial pressure, HCO_3_: bicarbonate, Cstat: static lung compliance, Cdyn: dynamic lung compliance, Raw: airway resistance, Ppic: peak Airway pressure, Paw: mean airway pressure. Average ± SD. P1 value shows the difference between Student’s t-test for related samples of the Steen group at baseline vs. endpoint while P2 value shows the difference between Student’s t-test for related samples of the LPDA group at baseline vs. endpoint. P3 value indicates the significant difference at the endpoint of the two groups by Student’s t-test for independent samples.

### Histological findings

**Fig. 1:**
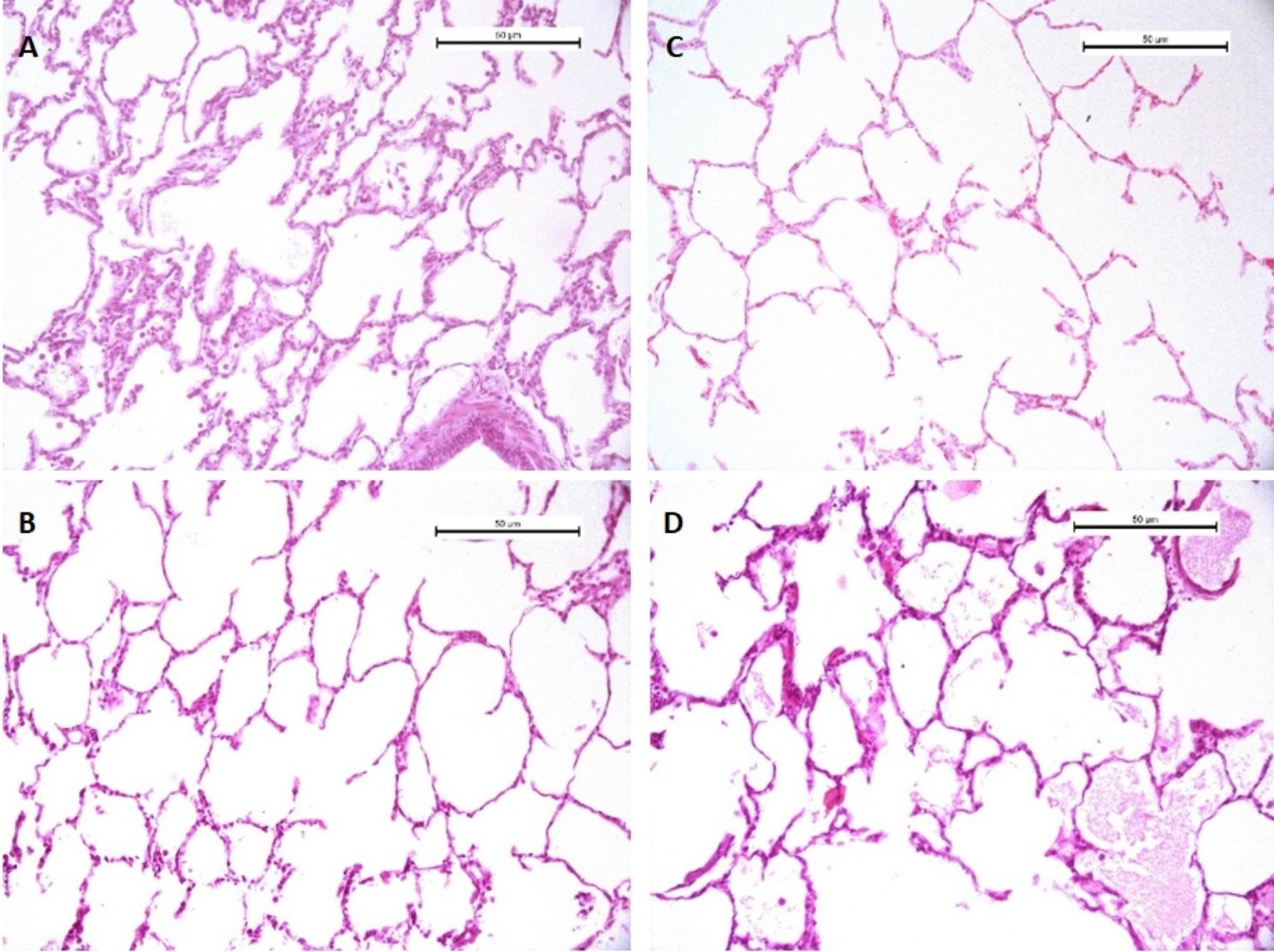
Shows histological images for the Steen and LPDA groups. It provides representative data obtained after treating the lungs with hematoxylin and eosin (H&E) staining. This figure illustrates the beginning and ending scores for both groups: Sections (**A**) and (**B**) for the Steen group and sections (**C**) and (**D**) for the LPDA group, respectively. Scale bar 20 μm

The lung injury severity score was better in Group I than in Group II (p < 0.05 Mann–Whitney U) with fewer neutrophils, macrophages, lymphocytes and edema; and the findings in Group 2 were statistically significant when compared to their baseline EVLP values (p < 0.05 Wilcoxon test). Table 2; Fig. 1.

**Table 2:** Histological features of the lungs

		Start	End	*P Value* ^*1*^
Lymphocytes	Steen	0.007 (0–0)	0.007(0–0)	1.000
	LPDA	0.007 (1 − 0)	0.007(1 − 0)	0.180
	P Value2	0.548	0.032	
Macrophages	Steen	0.007 (0–0)	0.007 (0–0)	0.157
	LPDA	0.007(1 − 0)	0.007(1–1)	0.180
	P Value2	0.548	0.016	
Neutrophils	Steen	0.007 (0–0)	0.007 (0–0)	1.000
	LPDA	0.007 (1 − 0)	0.007 (1 − 0)	0.180
	P Value2	0.421	0.016	
Edema	Steen	0.007 (0–0)	0.007 (0–0)	0.37
	LPDA	0.007 (0–0)	0.007 (0–0)	1.000
	P Value2	1.000	0.690	

Medians (interquartile range). *P Value*^1^ difference by Wilcoxon rank analysis of related samples. *P Value*^2^ significant difference between the two groups by means of the Mann-Whitney U test for independent samples

### Transmission electron microscopy

**Fig. 2:**
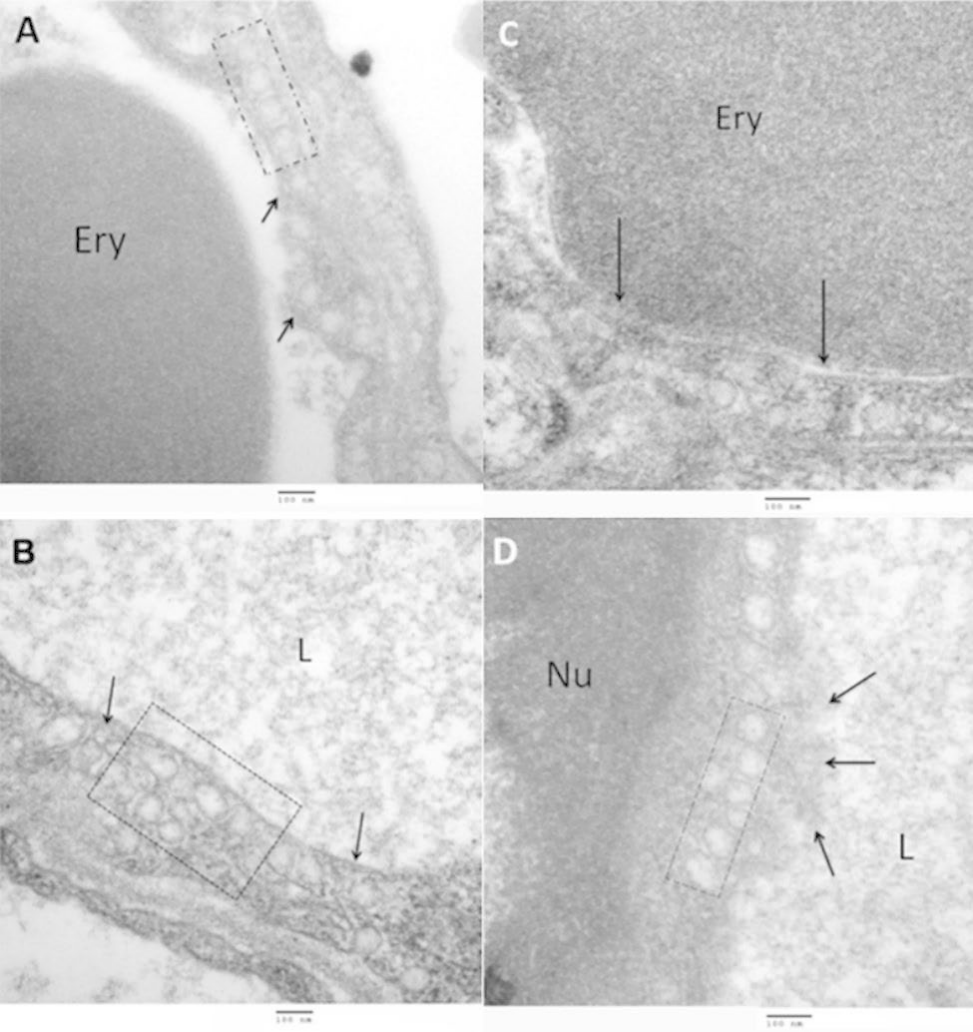
Transmission electron photography, uranyl acetate and lead citrate contrast technique. Close-up of an alveolar capillary in the Steen® solution group. (**A**) shows an erythrocyte (Ery) in the lumen. The endothelial cell surface displays short, discreet electrodense projections (arrows) suggestive of EG. (**B**) shows the lumen (L) occupied by slightly electrodense proteinaceous material. The box contains an image of pinocytic vacuoles in the cytoplasm (arrows) **C**) shows a bridge between an (Ery) and the completely smooth surface of an endothelial cell. (**D**) shows a close-up of an endothelial cell. The (L) is clearly occupied by slightly electrodense proteinaceous material. The endothelial cell surface displays short, discreet electrodense projections (arrows) suggestive of EG. The box contains an image of an endothelial cell cytoplasm with numerous pinocytic vacuoles. (Nu) endothelial cell nucleus

We obtained electron micrographs to determine the ultrastructural integrity of the lungs following EVLP. We reviewed ultrafine slices of the lungs divided into two samples: prior to (start) and at 120 min (end) of EVLP. Initially, the pulmonary lumen of both groups showed alveolar capillaries occupied by erythrocytes, with the surface of the endothelial cells being mostly smooth. Multifocally, some cells showed discreet electron-dense projections (suggestive of glycocalyx), mitochondria with normal morphology and pinocytic vesicles. Ultrastructurally, at 120 min after the procedure, the endothelial surface of the lungs showed discreet electrodense projections as well as pinocytic vacuoles and caveolae. Figure 2.

### Wet/Dry ratio

The wet/dry ratio (W/D) was higher in the LPDA (0.85 g ± 0.02 SD) than in the Steen (0.75 g ± 0.02 SD) group; however, the difference (p > 0.05) was not statistically significant.

### Immunohistochemical expression

**Fig. 3:**
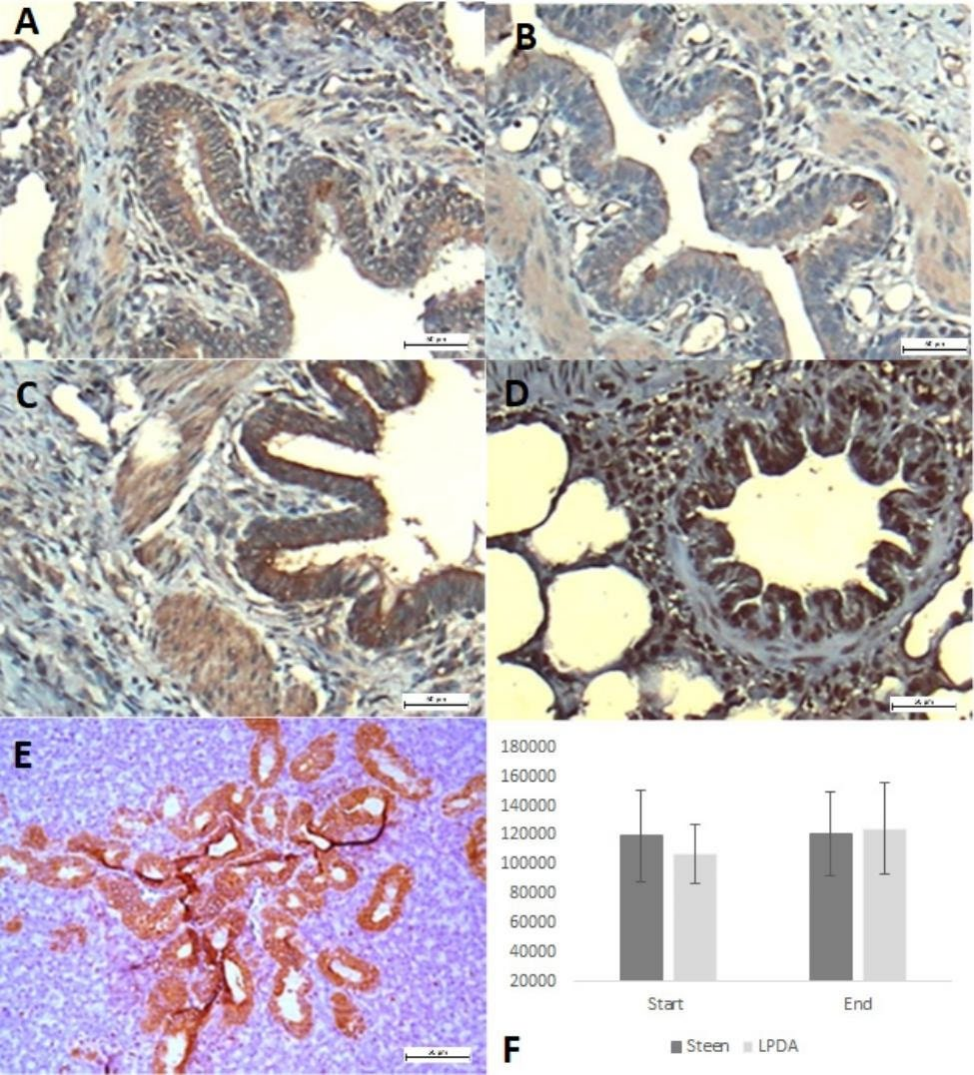
Lung tissue. Immunostaining with diaminobenzidine (DAB) (brown color). Positive immunolabeling in respiratory epithelium. **A**, **B**) Kinetics of 120 min of EVLP of group I Steen; and **C**, **D**) group LPDA, where positive immunolabeling is observed in the cytoplasm of the respiratory epithelium; **E**) salivary gland tissue used as positive control and F) shows the number of Sia-positive pixels in both study groups. Scale bar 20 μm

In both groups, 100% of the tissue samples subjected to EVLP showed SA expression in a diffuse cytoplasmic pattern on the luminal surface of blood vessels, mucosal lamina propria, bronchiole submucosa, and alveolar epithelial cells. There was no significant difference between groups (p = 0.860) or at the end of the study for Group 1 (p = 0.502) and Group 2 (p = 0.330). Figure 3.

**Fig. 4:**
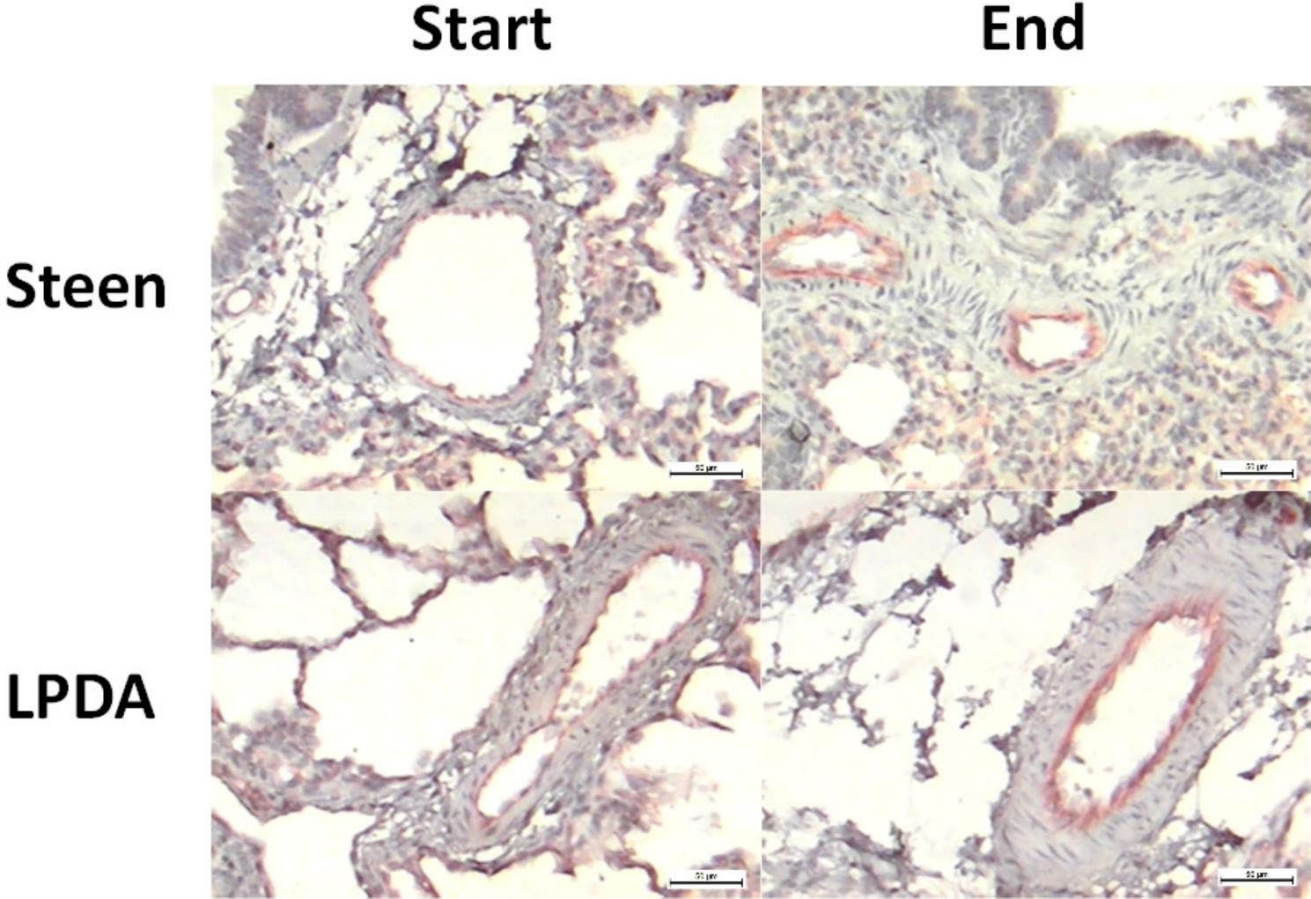
Example of CD31 staining showing increased positivity of blood vessels at the end of EVLP. Scale bar 20 μm

Clear vascular staining of PECAM-1 could be observed in the endothelial cells of both groups (p = 0.043, Wilcoxon signed-rank test) in Group I and (p = 0.039, Wilcoxon signed-rank test) Group II. With a higher expression of PECAM-1 at the end of EVLP. This difference was not statistically significant between groups (p = 0.15, Mann‒Whitney U test). Figure 4.

### Immunofluorescence findings

**SA-a-2,6-terminal saccharides (SNA lectin).** There was a signal coating of the SNA lectin on the epithelial cells of the bronchioles and alveoli. There were no differences in the SNA signal of the lung tissue between the different groups of pigs.

**SA-a-2,3-terminal saccharides (MAAII lectin).** The MAAII lectin signal demonstrating the SA-α-2,3-terminal saccharides was more pronounced than the SA lectin signal at the end of EVLP. MAAII was found in epithelial cells of alveoli and bronchioles. Figure 1.

**Fig. 5:**
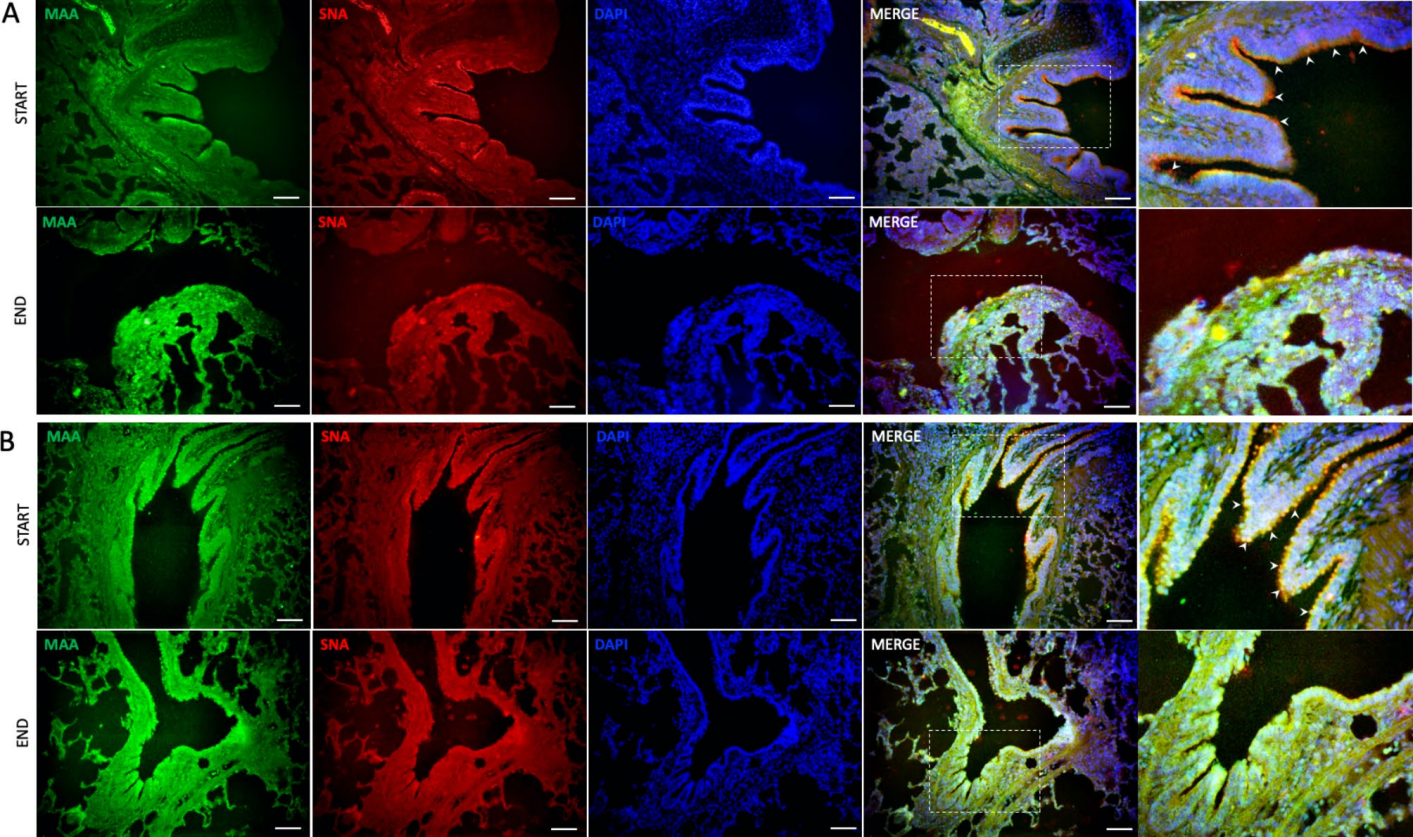
Expression of sialic acids in lung tissue. **A**. Group treated with Steen ®, **B**. Group treated with LPDA. Sambucus nigra agglutinin (SNA) to SAα2,6 and Maackia amurensis agglutinin (MAA) for binding to SAα2,3. Double fluorescence for TRITC labeled SNA and FITC labeled MAA, cellular nuclei counterstaining using DAPI. Arrow indicates sialic acid expression in the tissue. Scale bar 25 μm|

In addition, the connective tissue of the lamina propria and submucosa were MAAII positive. Figure 5.

### Levels of free Sia

**Fig. 6:**
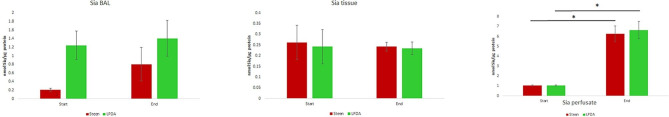
Shows Sia concentrations in the three sample types (BAL, tissue and perfusate) from both study groups at the beginning and end (at 120 min) of the EVLP procedure. Average ± SD *p < 0.05

We measured the levels of free Sia in three sample types and, as expected, detected Sia in all three samples. Similar concentrations were observed between groups, but compared to their initial values, Sia levels were increased to a lesser extent in the Steen® group in the bronchoalveolar lavage (BAL) (p = 0.19), pulmonary tissue (p = 0.85) and perfusate (p = 0.003). The LPDA group exhibited similar findings in the BAL (p = 0.77), lung tissue (p = 0.94), and perfusate (p = 0.003). Differences in Sia concentration were not statistically significant between groups. Figure 6.

When correlating free Sia levels in, lung tissue, and perfusate and BAL fluid, no significant differences were observed in any case: Tissue/ PVR (R² = -0.3101918, p = 0.3831); Perfusate/PVR (R² = 0.08735245, p = 0.8104; BAL/PVR (R² = 0.2841391, p = 0.4262). Tissue/raw (R²=0.552935, p = 0.09736); perfusate/raw levels (R²= 0.5696205, p = 0.08563); BAL/Raw (R² = 0.3078939, p = 0.3868). Tissue/Cstat (R² = -0.2604778, p = 0.4673); perfusate/Cstat (R² = -0.1875306, p = 0.6039); BAL/Cstat (R² = 0.119729; p = 0.7418). Tissue/W/d (R² = 0.2690493, p = 0.4522); perfusate/ W/d (R² = 0.1876282, p = 0.6037); BAL/ W/d (R² = 0.1434602, p = 0.6926).

## Discussion

General interest in the EVLP technique is increasing, and extensive studies have been conducted to investigate its potential. However, the question remains to what extent the platform and its standard solution can affect lung function and how it affects the interpretation of potential viability markers. Therefore, this study evaluated the influence of two ex vivo perfusion solutions on lung function and endothelial injury.

Our findings show that during 120 min of EVLP using a swine model, Sia levels were found in the BAL, lung tissue and perfusate in both study solutions during the first hour, with higher levels at the end of the study. Sia is an important component of the extracellular matrix in various tissues [18] and is involved in the biological functions of glycans, including their structural, cellular, physiological, and immunological functions [19].

In our study, both groups showed similar hemodynamic performance during EVLP assessment, with elevated PVR values for the LPDA group from baseline measurement, suggesting the persistence of scattered microthrombi despite the use of perfadex and heparin solution causing flow obstruction at the level of the small arterioles. [20]

Another consideration for this could be that during this process, the disruption of Sia, which regulates glycocalyx-mediated mechanotransduction [21, 22] and consequently vessel dilatation [23], could lead to endothelial cell dysfunction (ECD). EG components are essential for the maintenance of their function and the loss of anionic charges provided by Sia can lead to changes in the geometry of cellular clefts and direct endothelial injury [24, 25].

In accordance with the present results, previous studies have demonstrated that the three main components of glycocalyx—hyaluronic acid, heparan sulfate, and Sia—are involved in the induced production of endothelial NO in swine arteries ex vivo [23].

In a previous study, Sia was also shown to contribute to shear-induced NO production during perfusion of rabbit mesenteric arteries, and pretreatment with neuraminidase suppressed flow-dependent vasodilation [26]. Similarly, Hecker, et al. [27] found that when intact segments of rabbit femoral arteries were pretreated with neuraminidase, shear stress-induced NO production was inhibited. Along these lines, Zhang, et al. [28] showed that in mesenteric arteries perfused with degraded glycocalyx, the flow-induced vasodilator response was almost absent but was rapidly and completely restored when these vessels were perfused with nanoliposomal carriers of glycocalyx.

Consistent with our results, Meers, et al. [29] showed that subjecting injured swine lungs to EVLP stimulated an increase in PVR.

In addition to the increase in PVR, lung compliance decreased more markedly in the LPDA group, possibly because of impaired lung function. [3]. Our findings are consistent with those of Lowe, et al. [30] and Sanchez, et al. [20], who demonstrated that changes in pulmonary microcirculation directly affect the mechanical properties of the lung with a consequent increase in alveolar pressure compressing capillaries and increasing PVR. Therefore, the maintenance of controlled perfusion and ventilation are key factors in avoiding epithelial and endothelial injuries and the subsequent development of pulmonary edema during EVLP, and although lung-protective strategies were used in this study, the use of a roller pump could lead to the changes found in hemodynamics and pulmonary mechanics [11].

Mechanical ventilation during EVLP is usually performed by adapting the lung protection strategy used clinically in patients with acute respiratory distress syndrome. This approach, while reasonable, does not take into account the specific peculiarities of the isolated and perfused organ, compared to a whole organism. The absence of the thorax wall and diaphragm surrounding the lungs increases ex vivo the proportion of airway pressure acting directly on the lungs. [31] Santini A., et al., demonstrated that the lungs during EVLP, compared to in vivo, have lower current compliance, higher airway resistance, increased alveolar dead space, and higher gas content for the same airway pressure applied [31]. Therefore, airway pressures that are considered safe in patients could be detrimental to the lungs in the ex vivo environment. Despite its important contribution to lung reconditioning, this technique has the disadvantage that it can induce ventilator-induced lung injury [32].

Another complication in the evaluation of respiratory mechanics during the EVLP, is the repeated use of BAL that can cause heterogeneities in ventilation, that clearly impact on the general mechanical properties of the respiratory system including, of course, airway resistance and lung compliance. [33, 34]. The increase in Raw during EVLP can be suspected due to the presence of edema. [35]. However, it could have been associated with the decrease in both Cstat and Cdyn. Similarly, the increase in peak pressure suggests that when a certain degree of injury to the alveolocapillary membrane had occurred, with the subsequent formation of edema, additional pressure was required to distend the alveoli. The changes in Paw could have been associated with alterations in Pipc and positive end-expiratory pressure. These results are in agreement with Terragni, et al. [32], who observed that in lungs treated with EVLP for reconditioning prior to transplantation, some degree of mechanical ventilation-induced injury still occurs despite employing a lung-protective strategy.

ECD is characterized by increased vascular permeability, so the presence of edema in our study confirms the manifestations of ECD [28]. A possible explanation for these results is that high levels of Sia were present on the endothelial cell surface [36], transmitting the negative charge of the glycocalyx and regulating fluid displacement. Moreover, the loss of anionic charges could have led to changes in the geometry of the endothelial clefts, resulting in increased permeability [6, 19, 37]. However, our model found mild edema in both EVLP groups; in addition, we detected no deterioration of oxygenation until the final EVLP [38]. The PaO_2_/FiO_2_ quotient showed a slight increase compared to the initial EVLP values in both groups, which was most likely the result of the removal of atelectasis. Traditionally, PaO_2_/FiO_2_ quotients as a measure of pulmonary oxygenation capacity have proven to be the most accurate predictors of a successful lung transplant, and our results coincide with those reported by Cypel, et al. [39] and Stanzi, et al. [40].

Our histological findings showed a higher number of inflammatory cells in Group II than in Group I, probably because the ex vivo lung continues to be an important part of the generation of a strong inflammatory response since it harbors leukocytes in its alveolar and interstitial compartments [41]. Edema can occur as a consequence of the loss of the negative and hydrophilic charge of the endothelial surface. This process is determined by the presence of Sia, as has been previously described by several authors [4, 19, 41, 42]. Cioffi et al. [6] showed that treatment of endothelial monolayers with neuraminidase leads to disruption of the endothelial barrier in pulmonary artery endothelial cells and pulmonary vasculature. This led to the formation of interendothelial gaps and the disappearance of large areas of the monolayer, resulting in the loss of integrity of the endothelial barrier, liquids and proteins. However, it is still unknown whether a loss threshold for Sia must be reached for the interruption of the endothelial barrier to occur. Neither is it understood whether α (2,6) or α (2,3)-linked sialic acids, or both, are of critical importance for barrier integrity or whether acetylated sialic acids or (2,8) dimeric-linked sialic acids play a key role in the determination of such integrity.

In spite of the presence of edema, we detected no deterioration in the pulmonary structure, in line with what was reported by Medeiros, et al. [15] and Sadaria, et al. [43], all of whom found no deterioration in pulmonary tissue structure even after 12 h of normothermic perfusion. This was also consistent with other research findings that the pulmonary parenchyma was not altered in the majority of sections and showed extremely inflated alveolus, generally with thin alveolar septae; only a few sections had alveolus with edema. [38]

Our study found minimal changes in the ultrastructure of the lung samples from the EVLP process, in accordance with earlier observations showing that EVLP more effectively preserves functional pulmonary ultrastructure [38]. Although this conserved the glycocalyx in a multifocal manner in both study groups, its thickness was not measured, and we believe that the contribution of GE to hydraulic permeability was determined to a great extent by this characteristic. This was also noted by Betteridge, et al. [37], who confirmed an association between the disruption of the Sia and a reduction in EG thickness. This was sufficient to explain the increase in hydraulic conductivity after treatment with neuraminidase, suggesting that the GE must reach a minimal thickness to impede the flow of albumen. Our microscopic findings must be more solidly grounded, suggesting the need to undertake new comparisons of the ultrastructure, by employing setting techniques that do not significantly damage the electronegative structures, as is the case with Sia [44]. Nevertheless, we were able to generate detailed morphological results and compare them with functional findings.

Sialic acids are prominently expressed along the epithelial border lining the airways and are also major components of the secreted mucins in the airways [36]. Given that cellular sialylation is a dynamic process and that the balance between sialyltransferase and sialidase activity determines the state of sialylation on the cell surface [45], the inflammatory process could determine the increase in immunohistochemical expression at the end of EVLP. It is worth mentioning that to date, no study has reported the effect of sialylation during EVLP. However, studies have provided detailed insight into the functional consequences of increased cell membrane sialic acid in tumor cells [46] and have shown that the structure of cell surface α2–3 sialic acid could influence cell‒cell signal transduction and affect cell behavior [47].

On the other hand, we found higher expression of PECAM-1 in the lungs after EVLP than at the beginning of EVLP. Increased PECAM-1 levels could be caused by PECAM-1 upregulation in lung endothelial cells or by inflammatory cells, which supports data on endothelial dysfunction [48].

It is currently understood that glycocalyx degradation can occur during the EVLP procedure [49, 50]. Given the abundance and importance of Sia in key physiological processes, analyzing it in samples obtained during the EVLP process and understanding its possible relationship with pulmonary function may be important. We acknowledge the limitations of this study: first, there was a relatively small number of lungs available, which may also explain the inability to reach statistical significance in some parameters; second, unlike all previous studies assessing Sia, in our study, we were unable to determine total Sia levels. Nevertheless, we showed that Sia, as a degradation product of EG during the EVLP process, can be elevated at 120 min when using two different perfusion solutions, although there was no correlation with lung function.

## Data Availability

All data generated or analysed during this study are included in this published article.

## Abbreviations

EVLP: Ex vivo lung perfusion
EG: Endothelial glycocalyx
PG: Proteoglycans
Sia: Sialic acids
GAG: Glycosaminoglycans
Neu5Ac: N-acetylneuraminic acid
LPDA: Low-potassium dextran-albumin solution
PA: Pulmonary artery
PEEP: Positive end-expiratory pressure
PawP: Pulmonary artery wedge pressure
FiO_2_: Fraction of inspired oxygen
PVR: Pulmonary vascular resistance
PaFiO_2_: Pressure of oxygen and the fraction of inspired oxygen
PaO_2_: Partial pressure of oxygen
PaCO_2_: Partial pressure of carbon dioxide
HCO_3_: Bicarbonate
FiO_2_: Fraction of inspired oxygen
Cstat: Static lung compliance
Cdyn: Dynamic lung compliance
Raw: Airway resistance
Ppic: Peak inspiratory pressure
Paw: Mean airway pressure
H&E: Hematoxylin and eosin
TBST: Tris Buffered Saline, with Tween
DAB: 3,3′-Diaminobenzidine
IHC: Immunohistochemistry
NIH: National Institutes of Health
CD31/ PECAM-1: Platelet endothelial cell adhesion molecule 1
SNA: Sambucus nigra agglutinin
MAA: Maakia amurensis agglutinin
FICT: Fluorescein isothiocynate
TRITC: Tetrametyl rhodamine isothiocynate
TBS: Tris buffered saline
BAL: Bronchoalveolar lavage
Ery: Erytrocyte
ECD: Endothelial cell dysfunction
NO: Nitric oxide
